# Impact of birth weight and postnatal diet on the gut microbiota of young adult guinea pigs

**DOI:** 10.7717/peerj.2840

**Published:** 2017-01-03

**Authors:** Kait Al, Ousseynou Sarr, Kristyn Dunlop, Gregory B. Gloor, Gregor Reid, Jeremy Burton, Timothy R.H. Regnault

**Affiliations:** 1 Department of Microbiology and Immunology, University of Western Ontario, London, Ontario, Canada; 2 Canadian Centre for Human Microbiome and Probiotic Research, London, Ontario, Canada; 3 Lawson Health Research Institute, London, Ontario, Canada; 4 Department of Physiology and Pharmacology, University of Western Ontario, London, Ontario, Canada; 5 Department of Obstetrics and Gynaecology, University of Western Ontario, London, Ontario, Canada; 6 Children’s Health Research Institute, London, Ontario, Canada; 7 Department of Biochemistry, University of Western Ontario, London, Ontario, Canada; 8 Department of Surgery, Division of Urology, University of Western Ontario, London, Ontario, Canada

**Keywords:** Microbiome

## Abstract

**Background:**

The gastrointestinal tract (GIT) microbiota is essential to metabolic health, and the prevalence of the Western diet (WD) high in fat and sugar is increasing, with evidence highlighting a negative interaction between the GIT and WD, resulting in liver dysfunction. Additionally, an adverse *in utero* environment such as placental insufficiency resulting in low birth weight (LBW) offspring, contributes to an increased risk of metabolic diseases such as fatty liver infiltration and liver dysfunction in later life. We sought to understand the potential interactive effects of exposure to a WD upon growing LBW offspring. We postulated that LBW offspring when challenged with a poor postnatal diet, would display an altered microbiota and more severe liver metabolic dysfunction.

**Methods:**

The fecal microbiota of normal birth weight (NBW) and LBW young guinea pig offspring, weaned onto either a control diet (CD) or WD was determined with 16S rRNA gene next generation sequencing at young adulthood following the early rapid growth phase after weaning. A liver blood chemistry profile was also performed.

**Results:**

The life-long consumption of WD following weaning into young adulthood resulted in increased total cholesterol, triglycerides and alanine aminotransferase levels in association with an altered GIT microbiota when compared to offspring consuming CD. Neither birth weight nor sex were associated with any significant changes in microbiota alpha diversity, by measuring the Shannon’s diversity index. One hundred forty-eight operational taxonomic units were statistically distinct between the diet groups, independent of birth weight. In the WD group, significant decreases were detected in *Barnesiella, Methanobrevibacter smithii* and relatives of *Oscillospira guillermondii*, while *Butyricimonas* and *Bacteroides spp.* were increased.

**Discussion:**

These results describe the GIT microbiota in a guinea pig model of LBW and WD associated metabolic syndrome and highlight several WD specific GIT alterations associated with human metabolic disease.

## Introduction

Metabolic diseases such as obesity and the related metabolic syndrome are now considered to be an epidemic and an increasing burden on health care systems (Mathers et al., 2001). The gastrointestinal tract (GIT) microbiota is essential to metabolic health, and a dysfunctional GIT is closely linked to the development of aspects of metabolic syndrome. The GIT microbiota utilizes indigestible components of our diets and some suggest it may influence calorie harvesting from food (Turnbaugh et al., 2006; Zeng et al., 2013). It also has an important role in homeostasis and the maintenance of epithelial barriers, which when degraded may contribute to inflammation leading to chronic diseases characterized by metabolic dysfunction such as non-alcoholic fatty liver disease (NAFLD) and diabetes (Bäckhed et al., 2004; Dunne et al., 2014).

Due to the divergent nutritional requirements of various bacteria residing in the gut, diet has been shown to shape the composition of the microbiota, which in turn may lead to adverse health outcomes such as metabolic syndrome (Turnbaugh et al., 2008; Turnbaugh et al., 2009). Specifically, the consumption of a typical “Western” diet (WD) high in fat and sugar has been shown by some groups to alter the microbial diversity and relative abundance of two main phyla in humans and mice, *Bacteroidetes* and *Firmicutes* (Turnbaugh et al., 2009). For these reasons, the gastrointestinal microbiota is considered one of the potential environmental factors that advance the host to a metabolically diseased state (Hildebrandt et al., 2009).

An emerging factor potentially regulating the GIT microbiota composition is early life conditioning through pregnancy and during early postnatal life. While it is not yet clear how an adverse *in utero* environment specifically impacts the new born microbiota, studies report that placental insufficiency outcomes are associated with an altered neonatal GIT and caecocolonic microbiota, an alteration that in some reports continues into later life (Trahair et al., 1997; Sangild, Fowden & Trahair, 2000; Fança-Berthon et al., 2010; Yan et al., 2011). This altered gut flora is associated in animal and human studies with failure of adequate postnatal growth (Trahair et al., 1997; Yan et al., 2011). In support of these observations, gut microbiota modulation by diet, prebiotics, or probiotics may modify the growth pattern of the offspring or prevent the development of adverse *in utero* environment-induced diseases (Luoto et al., 2010; Arrieta et al., 2014). In addition to modulating the new born gut composition, the *in utero* environment, resulting in a reduced fetal growth trajectory, plays a major role in setting the offspring’s risk of metabolic disease later in life (Browne, 1962; Barker et al., 1993; Barker, 2000; Yan et al., 2011). This is referred to as the “thrifty hypothesis”, whereby low birth weight (LBW) offspring experience permanent changes in their metabolic function *in utero*, which are determinant in later postnatal life when challenged with nutrient excess (Thorn et al., 2011). These metabolic abnormalities include fatty infiltration of the liver and liver dysfunction highlighted by elevated alanine aminotransferase (ALT) levels (Angulo et al., 1999; Hales & Barker, 2001).

Guinea pigs have been used interdependently in the study of *in utero* growth, fetal development, and the impact diet has on postnatal growth (Fernandez & Volek, 2006; Sarr et al., 2014; Sarr et al., 2015; Thompson et al., 2014). A limited number of studies have described the guinea pig intestinal microbiota and have highlighted an overlap of phyla present in both the guinea pig and human GIT (Yanabe et al., 2001; Takahashi et al., 2005; Hildebrand et al., 2012). The aims of the present pilot study were to determine whether an *in utero* environment resulting in LBW is a factor in the compositional development of the gut and hepatic manifestations of metabolic syndrome, specifically altered ALT, and to investigate how a WD may impact these outcomes in growing offspring.

## Materials and Methods

### Ethics statement

Animal care, maintenance, and surgeries were conducted in accordance with the standards set by the Canadian Council on Animal Care. The University of Western Ontario Animal Use Subcommittee approved all procedures (AUP # 2010-229).

### Animals and diets

Time-mated pregnant Dunkin-Hartley guinea pigs (Charles River Laboratories, Wilmington, MA, USA) were housed in a temperature (20–22 °C) and humidity (30%) controlled environment with a 12 h light–dark cycle and had access to chow and tap water provided *ad libitum*.

Chow-fed pregnant guinea pigs underwent uterine artery ablation (UAA) surgery at mid gestation (∼32 days, term 69 days) to generate normal and low birth weight offspring (NBW and LBW, respectively) due to chronic placental insufficiency as described previously (Turner & Trudinger, 2009; Sarr et al., 2014; Thompson et al., 2014). Sows delivered spontaneously at term (∼67 days) and birth weight was recorded. Guinea pig pups from a UAA pregnancy weighing less than 85 grams were defined as LBW, and pups weighing 90 grams or greater at birth were defined as NBW (Elias et al., 2015). Five days prior to weaning the postnatal control diet (CD, TD: 110240; Harlan Laboratories, Madison, WI, USA) was introduced to the pups through the maternal feeding tray. At 15 days of age the offspring were weaned, separated by sex, weighed, housed in individual cages, and randomized to either CD or a Western diet (WD, WD: 110239; Harlan Laboratories), as described previously (Thompson et al., 2014). Briefly, the diets differed in kilocalorie density (3.4 vs 4.2 kcal g^−1^), but were matched for protein and macronutrients. The percentage of kilocalories for CD and WD from protein was 21.6 and 21.4, from fat was 18.4 and 45.3, and from carbohydrates was 60 and 33.3. Additionally, the WD contained 2.5 g kg^−1^ cholesterol. To avoid litter effects, only one LBW/NBW animal per sex from a single litter was assigned to each diet. From the time of weaning, food intake was recorded daily until sacrifice by CO_2_ inhalation at young adulthood ∼150 days. At sacrifice, blood was collected to quantify total cholesterol and triglyceride levels, as well as to conduct a liver blood chemistry profile (ALB, ALP, ALT, BA, BUN, GGT, and TBIL) using a Vetscan VS2 (Abaxis, Union City, CA). Fecal samples were also collected at sacrifice by emptying colon contents into a sterile bag, then immediately stored at −80 °C until further analysis.

### Fecal DNA extraction

The MoBio PowerSoil^®^  96-Well Soil DNA Isolation Kit (Mobio, Carlsbad, CA), was used according to the modified Earth Microbiome Project standard protocols (Earth Microbiome Project, 2016). Approximately 0.25 g of each fecal sample was transferred to each well using sterile pipette tips, and extracted DNA was stored sealed at −20 °C until PCR.

### Fecal sample polymerase chain reaction

Fifty microlitres of the DNA template extract was transferred to a 96-well PCR plate (Axygen, Union City, CA). The BioMek^®^ 3000 Laboratory Automation Workstation was used for automated PCR reagent set up. Amplifications of the V4 region of the 16S ribosomal RNA gene were carried out with the primers ACACTCTTTCCCTACACGACGCTCTTCCGATCTNNNNxxxxxxxxGTGCCAGCMGCCGCGGTAA and CGGTCTCGGCATTCCTGCTGAACCGCTCTTCCGATCTNNNNxxxxxxxxGGACTACHVGGGTWTCTAAT wherein xxxxxxxx is a sample specific nucleotide barcode and the preceding sequence is a portion of the Illumina adapter sequence for library construction. Ten microlitres (2.3 pmol/µl) each of a total of 32 primers, 16 left and right with unique barcodes were arrayed in 96 well plates. Using a BioMek 3000^®^ (Beckman Coulter, Brea, CA, USA) 2µl of the DNA template was transferred into a plate containing 10 µl of each unique primer. Then 20 µl of Promega GoTaq^®^ Colourless Master Mix (Promega, Maddison, WI, USA), containing the necessary dNTPs, PCR reaction buffer, MgCl_2_, and GoTaq^®^ DNA Polymerase was added to the DNA template and primers. The final plate was firmly sealed with a foil PCR plate cover. This plate was placed in the Eppendorf Mastercycler^®^ thermal cycler (Eppendorf, Mississauga, ON), where the lid was kept at 105 °C. An initial hot start temperature of 95 °C was used for two minutes to activate the GoTaq^®^. This was followed by 25 cycles of 95 °C for one minute, 50 °C for one minute, and 72 °C for one minute. After completion, the reaction was held at 4 °C until collection and then the amplicons were stored at −20 °C.

**Table 1:** Animal characteristics and metadata.

	Control diet		Western diet		Diet	Birth weight	Interaction
	NBW^a^ (*n* = 9)	LBW^b^ (*n* = 10)	NBW (*n* = 6)	LBW (*n* = 8)			
Distribution of Sex	F:4/M:5	F:5/M:5	F:2/M:4	F:5/M:3			
Body weight (g)	752.13 ± 36.00	648.46 ± 42.15	704.45 ± 27.9	585.99 ± 46.59	NS^c^	*P* < 0.05	NS
Daily energy intake (kcal)	149.94 ± 5.48	192.98 ± 22.16	213.92 ± 9.40	210.81 ± 19.17	*P* < 0.05	NS	NS
Liver Triglycerides	3.92 ± 0.74	5.51 ± 1.08	68.40 ± 11.39	75.74 ± 14.20	*P* < 0.0001	NS	NS
Blood analysis							
ALB^d^	4.04 ± 0.10	3.50 ± 0.41	4.20 ± 0.17	4.33 ± 0.17	*P* < 0.05	NS	NS
ALP^e^	62.40 ± 11.30	43.00 ± 8.50	65.14 ± 7.67	76.67 ± 7.56	NS	NS	NS
ALT^f^	49.20 ± 3.11	49.00 ± 5.58	94.57 ± 16.40	125.50 ± 32.57	*P* < 0.05	NS	NS
BA^g^	58.80 ± 21.26	46.00 ± 19.09	59.43 ± 10.19	63.33 ± 16.67	NS	NS	NS
BUN^h^	27.80 ± 35.60	26.75 ± 4.77	33.57 ± 4.35	28.83 ± 2.01	NS	NS	NS
TBIL^i^	0.05 ± 0.05	0.20 ± 0.00	0.23 ± 0.04	0.10 ± 0.06	NS	NS	*P <* 0.05
Cholesterol	77.00 ± 14.08	72.50 ± 15.18	418.00 ± 41.07	449.67 ± 30.41	*P* < 0.0001	NS	NS

**Notes.**

a NBW, Normal birth weight, or >90 g.

b LBW, Low birth weight, or <85 g.

c NS, Not significant.

d ALB, Albumin.

e ALP, Alkaline Phosphatase.

f ALT, Alanine Aminotransferase.

g BA, Bile Acids.

h BUN, Blood Urea Nitrogen.

i TBIL, Total Bilirubin.

Values are represented as mean ± the standard error of the mean. Significance tests were performed using 2-way ANOVA.

### DNA sequencing and data analysis

Samples were sent to the London Regional Genomics Centre at Robarts Research Institute (Western University, London, ON, CAN), where the sample quantification, clean-up, and sequencing were also performed. Amplicons were quantified using Picogreen (Quant-It; Life Technologies, Burlington, ON, CAN) and pooled at equimolar concentrations before cleanup (QIAquick PCR clean up; Qiagen, Germantown, MD, USA). The final samples were sequenced using the MiSeq by Illumina^®^ platform, with 2 × 300 bp paired-end chemistry. Obtained reads were quality filtered and overlapped using USEARCH including reads with one or fewer sequencing errors, and binned into OTUs based on 97% identity (Edgar, 2010). Statistical significance in animal characteristics and hematological analysis was determined using 2-way ANOVA (GraphPad Software, San Diego, CA, USA). Diversity analysis was performed using the R package Vegan (version 2.3-2), differential abundance analysis was performed using the R package ALDEx2 (version 1.4.0) and all additional analysis was performed in base R (version 3.2.2). Utilized scripts are provided in Data S7 and demultiplexed reads are available in the NCBI Sequence Read Archive: BioProject ID PRJNA344687 (Edgar, 2010; Fernandes et al., 2013; Fernandes et al., 2014; R Development Core Team, 2008).

**Figure 1:**
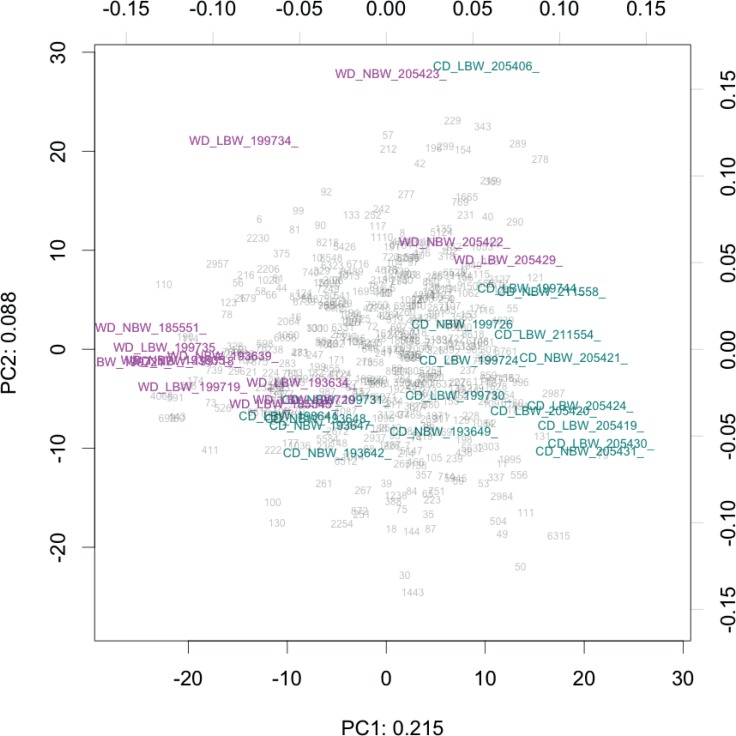
Compositional biplot of all samples. Samples are coloured according to diet, OTUs are shown as grey numbers. Approximately 30% of the variance is explained in the first two components. The biplot is drawn to show the relationship between the samples (scale = 0), as opposed to OTUs.

## Results

### Physical and hematological analysis of animal groups

The characteristics of each animal feeding group are displayed in Table 1. Animals body weights at sacrifice were lower in the LBW group (*p* = 0.011), but were not significantly different between the diet groups, despite the differences in the diets’ nutritional compositions. Both daily caloric intake and liver triglycerides were significantly elevated in the WD group (*p* = 0.027 and *p* < 0.0001). Liver blood chemistry profiles revealed no difference in alkaline phosphatase, bile acids, or blood urea nitrogen. Diet, but not birth weight, was a significant factor where WD groups had higher albumin, alanine aminotransferase, and cholesterol than CD groups (*p* = 0.031, *p* = 0.011, *p* < 0.0001, respectively). There was a significant diet and birth weight interaction in total bilirubin (*p* = 0.024).

**Figure 2:**
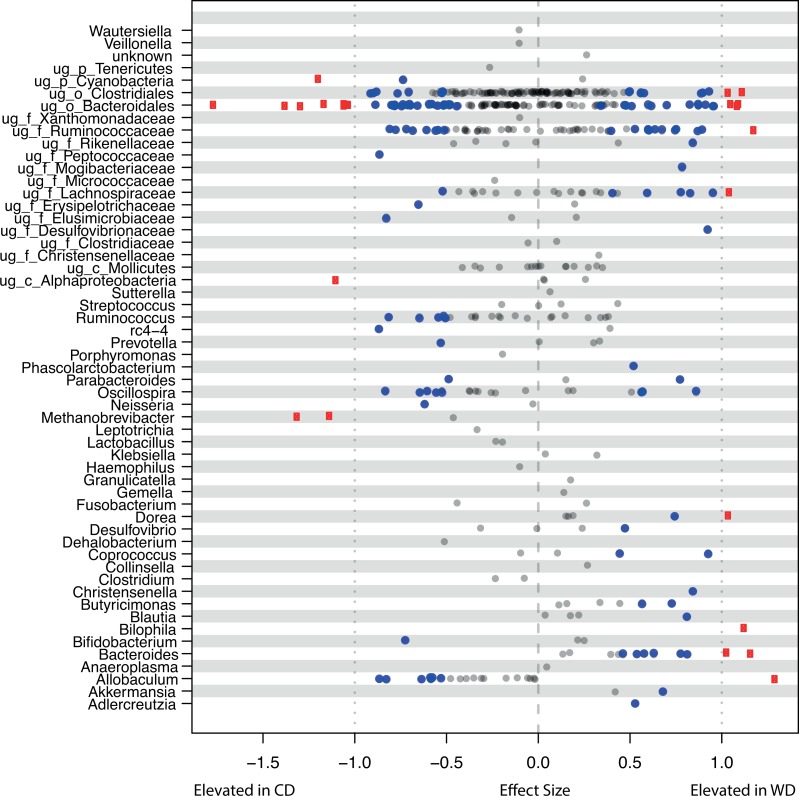
Stripchart of differential OTUs between diet groups. OTUs with a Benjamini–Hochberg corrected *p*-value from Wilcoxon rank-sum test <0.1 are plotted in blue. OTUs with *p* < 0.1 and an absolute effect size >1 are red. OTUs are summarized to genus. If genus is unknown (ug_), the lowest known taxonomic rank is stated (f_Clostridiaceae).

### 16S rRNA gene sequence-based characterization of the guinea pig fecal microbiota

Bacterial DNA amplified from fecal samples was grouped by 97% sequence similarity and assigned to a taxonomy using the Ribosomal Database Project Classifier (Wang et al., 2007). The sequences were grouped into 11 phyla, 19 classes, 29 orders, 45 families, and 73 genera, after filtering for OTUs representing 0.1% in any sample (data in Data S1). The Shannon’s index was used to measure the diversity of the individual samples (Shannon, 1948; Haegeman et al., 2013) and, surprisingly, a 2-way ANOVA did not detect a statistically significant effect of diet or birth weight groups (Fig. S1A, Data S2). Similarly, there were no significant differences between the mean number of reads per sample (Fig. S1B).

Principal component analysis (PCA) of centered log-transformed distances highlighted differences in the microbiota samples between the CD and WD fed animals (Fig. 1) (Van den Boogaart & Tolosana-Delgado, 2013). Distance on this plot represents overall dissimilarity between the microbiota profiles of the samples using the Aitchison distance which is appropriate for compositional data (Van den Boogaart & Tolosana-Delgado, 2013). The distance of each OTU from the centre of the plot is proportional to the standard deviation in the dataset (up to the limit of the projection displayed). Comparing Aitchison distance, microbiota profiles did not cluster on the plot by sex or birth weights of the animals, but did cluster distinctly when the animals were grouped by diet type (Fig. 1, Fig. S2). Diet groups were significantly different (*p* < 0.1) for 148 OTUs, when tested using the R package ALDEx2 with the non-parametric Wilcoxon rank-sum test with false discovery rate correction (Data S3, Fig. S3). The most significant of the 148 is OTU 22, and is most closely related to the genus *Barnesiella* from the family *Porphyromonadaceae* (*p* = 1.08 × 10^−4^) which was decreased in the WD animals. Other notable OTUs include the Archaea *Methanobrevibacter* (OTU 11 and 6160) and *Oscillospira* (OTU 77) which were reduced in the WD animals, while the genera *Bacteroides* (OTU 26 and 51), *Bilophila* (OTU 26), *Coprococcus* (OTU 165), and *Desulfovibrio* (OTU 76) were enriched in the same group. Figure 2 illustrates the differential OTUs between the diet groups and shows how individual OTUs from the same lineage behave. OTUs are plotted by effect size (the median of the ratio of the between diet group difference and the largest of the variance within groups) and Benjamini–Hochberg corrected Wilcoxon rank-sum test.

The most abundant phyla in all samples were *Bacteroidetes* and *Firmicutes*. Diet had a suggestive but not significant effect increasing the relative proportion of *Bacteroidetes* (*p* = 0.0832) in the WD group. The relative proportion of *Firmicutes* was significantly lower in the WD group compared to control (*p* = 0.0049). Birth weight had no effect on the relative proportions of either phylum.

## Discussion

This was a pilot study investigating the gut microbiota in an established guinea pig model of metabolic syndrome. The model utilizes a combination of uterine artery ablation to induce LBW offspring, with a postnatal diet high in total fat and sugar, and produces a non-overweight phenotype with impaired vascular function, increased visceral adiposity, and liver fibrosis with fatty infiltration of the liver, hallmarks of metabolic disease (Sarr et al., 2014; Sarr et al., 2015; Thompson et al., 2014).

In the current study, birth weight was not significantly associated with an altered GIT microbiota. However, a change in GIT microbiota was observed as a function of the animals’ diet, an effect strong enough to possibly overshadow any potential influence of birth weight on the microbiota. Alterations in the relative abundance of specific OTUs in the guinea pig GIT are in agreement with both human studies and other animal models. For example, the genus *Bacteroides* was significantly higher in the WD group, and is observed to be elevated in overweight women, while the genus *Methanobrevibacter* and relatives of *Oscillospira guillermondii* have been associated with low BMI in humans and were both comparatively decreased in the WD group (Collado et al., 2008; Million et al., 2011; Million et al., 2013). OTU 22 is most closely related to the genus *Barnesiella*, and was the most significantly different between our diet groups. *Barnesiella* has previously been shown to be increased in a non-obese diabetic rat model, but was decreased in our WD group (Zened et al., 2012; Marietta et al., 2013). Interestingly, this organism has been shown in rodents to be a marker of health as it assists in the clearance of less desirable bacterial colonization following antibiotic use, and is important in microbiome restoration (Ubeda et al., 2013). Similarly, this bacterial group may be outcompeted in our WD group but act as a marker of health in the CD animals.

When analyzing the data at the phylum level, it was observed that the guinea pig fecal microbiota is dominated by *Bacteroidetes* and *Firmicutes* in all diet and birth weight groups, similar to other rodent reports (Eckburg et al., 2005; Hildebrand et al., 2012). It was interesting to note that the prototypical decrease in relative abundance of *Bacteroidetes* accompanied by an increase in *Firmicutes* captured through a “*B*∕*F*” ratio as observed by some in obese or metabolic syndrome studies, was not observed in our WD group (Ley et al., 2006; Turnbaugh et al., 2006; Furet et al., 2010). Diet and birth weight did not have any effect on the relative proportion of *Bacteroidetes*, but the proportion of *Firmicutes* was decreased in the WD group compared to the CD group. This is not the first study unable to replicate the “stereotypical” shift observed between *B*/*F* phyla. Indeed, a recent meta analysis concluded that the ratio of *Firmicutes* and *Bacteroidetes* is not a consistent feature when comparing human obese and lean gut microbiota (Walters, Xu & Knight, 2014). Other factors contributing to the current study’s lack of *B*∕*F* change may be that guinea pigs are herbivorous and undergo hindgut fermentation, or that changes in *B*/*F* present at a later life stage than what was investigated herein (Duncan et al., 2008; Ley et al., 2008; Schwiertz et al., 2009; Walters, Xu & Knight, 2014). We caution that the convention of using the ratio of *B*∕*F* as a marker of the microbiome in metabolic disease may not be suitable for all animal models, especially in studies investigating the animal’s native microbiota as opposed to animals colonized by the human microbiota. Functional metagenomics, or reporting changes in particular genera and species, are likely to provide more insight (Hildebrand et al., 2012).

Alanine aminotransferase is one of the most commonly used markers in screening for liver disease, and levels were elevated in our WD fed animals independent of birth weight (Miyake et al., 2012). It is known that the GIT microbiota is a major factor in shifting the host to a metabolically diseased state, and the WD fed groups not surprisingly displayed elevated ALT levels and altered microbial markers, many of which are observed in humans with metabolic syndrome (Ley et al., 2006; Tims et al., 2012; Zened et al., 2012; Marietta et al., 2013; Ubeda et al., 2013). If diet is predominantly shaping the GIT microbiota, undesirable microbial products associated with WD may be translocating to the liver via the portal vein, impacting the liver function (Moore et al., 1991; Ilan, 2012; Hu et al., 2016). This can further induce hepatic tissue injury via activation of the inflammasome and chemokine release, creating a vicious circle of liver dysfunction (Ilan, 2012).

This study reports the WD-related changes to the gut microbiota of non-overweight young-adult guinea pigs with signs of early metabolic dysfunction (Sarr et al., 2014). The *in utero* environment resulting in low birth weight and metabolic disease at young adulthood appeared to have no impact upon the GIT microbiota, contrary to other reports in rats (Fança-Berthon et al., 2010). This lack of birth weight associated GIT changes at the age studied was in contrast to the driving role that diet appeared to play. A large number of OTUs identified by partial 16S rRNA gene sequence analysis were significantly different based on diet. Since changes were not largely detected in response to birth weight, if the microbiome does have a role here it may be occurring on a subtler basis rather than a global microbial shift. It is also possible the dietary effect on the microbiota was overshadowing any birth-weight related effects. An increase in the relative proportion of *Firmicutes* and decrease in *Bacteroidetes* was not observed in our WD group, and microbial diversity was largely unchanged. Despite this, several differential OTUs reported in the guinea pig associated with elevated ALT are also reported to occur in association with human metabolic disease. These observations highlight the potential usefulness of the guinea pig in understanding the negative impact of a diet high in saturated fats and sugar upon the GIT and its possible contribution to the development of metabolic disease.

##  Supplemental Information

10.7717/peerj.2840/supp-1 Data S1 OTU table OTUs were filtered to 0.1% in any sample.

10.7717/peerj.2840/supp-2 Figure S1 Shannon’s diversity index and read counts for sample groups Shannon’s diversity and read counts are not different between birth weight or diet groups.

10.7717/peerj.2840/supp-3 Data S2 Shannon’s diversity of every sample Output from diversity function in R (Vegan 2.3-2) performed on OTU table.

10.7717/peerj.2840/supp-4 Figure S2 Compositional biplot The samples are coloured according to diet and birth weight groups. The biplot is drawn to show the relationship between the OTUs [scale = 1].

10.7717/peerj.2840/supp-5 Data S3 ALDEx2 table Output from ALDEx2 test for significantly different OTUs between diet groups.

10.7717/peerj.2840/supp-6 Figure S3 ALDEx2 test heat map OTUs divergent between diet groups by Wilcoxon rank-sum test are shown. Colour corresponds to the log2 of the OTUs’ abundance.

10.7717/peerj.2840/supp-7 Data S4 Scripts utilized to generate figures R scripts are provided here to generate figures and data 1, 2, S2, S3, S4, S5, S6 from the provided OTU table (S1)

## Additional Information and Declarations

### Data Availability

The following information was supplied regarding data availability: The raw data has been supplied as a Supplemental File.
