# Supplementary figures and images for: Impact of birth weight and postnatal diet on the gut microbiota of young adult guinea pigs
Source: PeerJ. 2017 Jan 3;5:e2840. doi: 10.7717/peerj.2840 (PMC5214890; doi:10.7717/peerj.2840)

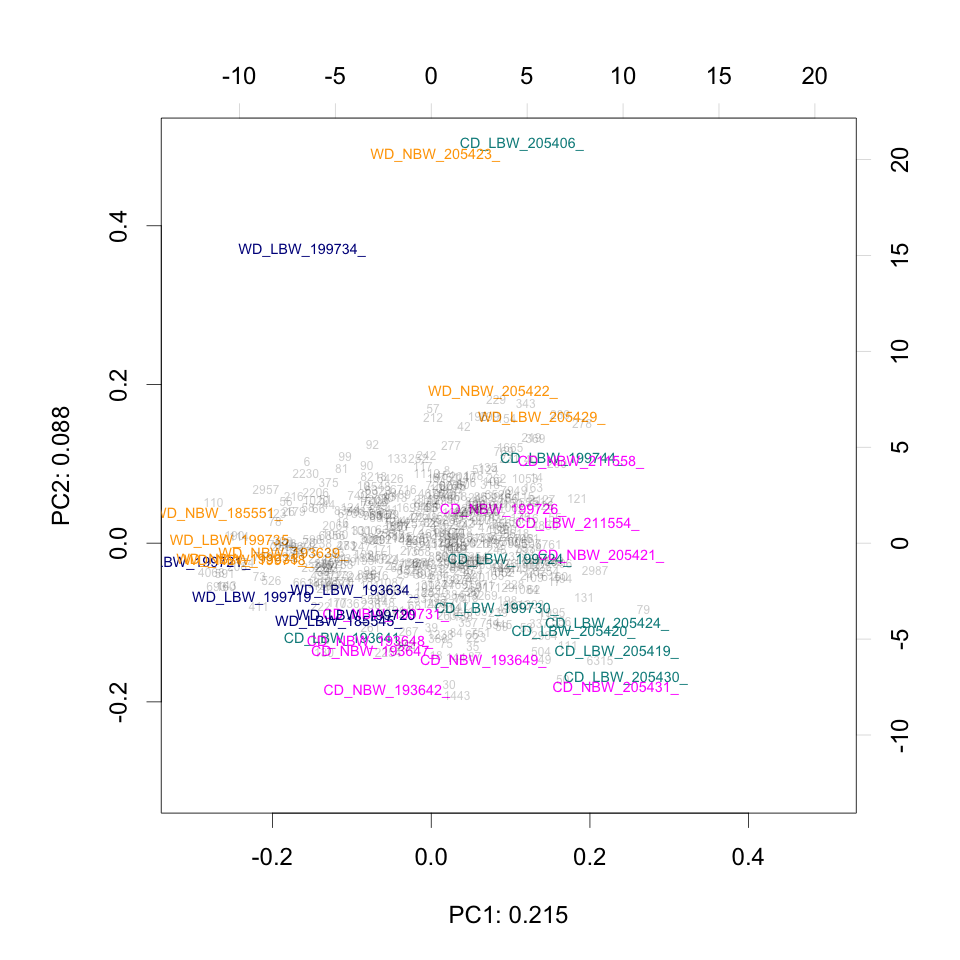

Supplement: Figure S2 — The samples are coloured according to diet and birth weight groups. The biplot is drawn to show the relationship between the OTUs [scale = 1]. [file peerj-05-2840-s004.png]
